# The practice effect of smartphone-derived cognitive processing speed assessments as a proxy of cognitive functioning in multiple sclerosis

**DOI:** 10.1007/s00415-026-13835-9

**Published:** 2026-05-07

**Authors:** Daan J. de Jong, Pam C. G. Molenaar, Vera Verhoef, Lenka Novakova, Elisa Colato, Tommy A. A. Broeders, Samantha Noteboom, Ka-Hoo Lam, Sonja Cloosterman, Bastiaan Moraal, Dennis Nieuwkamp, Oliver Gerlach, Jop Mostert, Eva M. M. Strijbis, Menno M. Schoonheim, Joep Killestein, Tom A. N. Fuchs

**Affiliations:** 1 MS Center Amsterdam, Amsterdam UMC Location Vrije Universiteit Amsterdam, Anatomy & Neurosciences, De Boelelaan 1117, Amsterdam, The Netherlands; 2 Amsterdam Neuroscience, Neuroinfection & Neuroinflammation, Amsterdam, The Netherlands; 3 MS Center Amsterdam, Amsterdam UMC Location Vrije Universiteit Amsterdam, Neurology, De Boelelaan 1117, Amsterdam, The Netherlands; 4 Department of Clinical Neuroscience, Institute of Neuroscience and Physiology, Sahlgrenska Academy, University of Gothenburg, Gothenburg, Sweden; 5 Department of Neurology, Region Västra Götaland, Sahlgrenska University Hospital, Gothenburg, Sweden; 6 MS Sherpa B.V., Nijmegen, The Netherlands; 7 MS Center Amsterdam, Amsterdam UMC Location Vrije Universiteit Amsterdam, Radiology, De Boelelaan 1117, Amsterdam, The Netherlands; 8 Department of Neurology, Jeroen Bosch Hospital, ‘s-Hertogenbosch, The Netherlands; 9 Department of Neurology, Academic MS Centrum Zuyd, Zuyderland MC, Sittard-Geleen, The Netherlands; 10 Department of Neurology, School for Mental Health and Neuroscience, Maastricht University Medical Center, Maastricht, The Netherlands; 11 Department of Neurology, Rijnstate Hospital Arnhem, Arnhem, The Netherlands; 12 MS Center Amsterdam, Neurology, Vrije Universiteit Amsterdam, Amsterdam Neuroscience, Amsterdam UMC Location VUmc, PO Box 7057, 1007MB Amsterdam, The Netherlands; 13 ReMuS, The Czech Republic Multiple Sclerosis Patient Registry, Prague, Czechia

**Keywords:** Multiple sclerosis, Cognition, Practice effects, Mobile devices

## Abstract

**Background:**

In multiple sclerosis (MS), symbol digit modalities test (SDMT) scores are often influenced by practice effects. We evaluated the SDMT practice effect as a proxy of cognitive performance by relating it to disease severity and future performance.

**Methods:**

People with MS (pwMS) and healthy controls (HCs) were evaluated at baseline and five-year follow-up. Practice effects were modeled using two-part piecewise linear regression on daily smartphone SDMT (sSDMT) scores. Cognitive impairment (CI) and preservation (CP) were defined relative to HC baseline sSDMT *z*-scores (CI: *z* < −1.67, CP: *z* ≥ −1.67). Practice outcomes across HC/CP/CI were compared using ANCOVA and correlated to baseline variables. They were also assessed in relation to clinical outcomes and brain volumes in cross-sectional and baseline–follow-up models.

**Results:**

85 pwMS (CP/CI: 66/19) and 20 HCs were analyzed. 73 pwMS completed follow-up (5.39 ± 0.38y). A practice plateau occurred in 80%/82%/100% of HC/CI/CP. Higher baseline sSDMT was related to higher plateau sSDMT (*ρ* = 0.930, *p* < 0.001) and lower %-increase (*ρ* = −0.266, *p* = 0.013). %-increase was higher in CI than CP (CI/CP = 22.0%/15.5 adj.*p* = 0.004), but the absolute ∆-increase and breakpoint were similar across groups. Associations of disability, cognition, and brain volumes with the plateau sSDMT were stronger when compared to the baseline sSDMT. No other associations were found cross-sectionally and in the baseline-follow-up models.

**Conclusions:**

Early-phase sSDMT practice effects were related to cognitive performance but were unique to disease status or associated with disease severity in pwMS. Plateau sSDMT showed stronger associations with disability and brain volumes than baseline performance. Interpretation of SDMT performance should therefore consider practice effects.

**Supplementary Information:**

The online version contains supplementary material available at 10.1007/s00415-026-13835-9.

## Introduction

Cognitive impairment (CI) is a common phenomenon in multiple sclerosis (MS) and can occur in any stage of the disease [1]. Cognitive information processing speed is the most commonly and earliest impacted ability [1–3], and can be assessed with the Symbol Digit Modalities Test (SDMT) [1,4]. The SDMT is subject to a practice effect: with repeated testing a gradual increase is observed over time [5]. Recent work in persons with MS (pwMS) showed that an average increase of 29% can be observed with many (18 or more) test repetitions﻿ and a plateau is reached after these many repetitions [6]. While efforts are employed to reduce this effect [7–9], as it may influence interpretation of cognitive testing [10, 11], some argue the effect should be regarded as biomarker of cognitive performance [12, 13, 8, 14, 15, 16]. Recent evidence from studies with variable time frames indicated that SDMT practice effects appear to be less pronounced in pwMS with more advanced disease [17, 14] and with more brain atrophy [14], and that the degree of the practice effect might predict future cognitive performance in MS and other neurologic diseases [12, 13, 8, 15].

Given the heterogeneous nature of study designs, additional evidence is required to conclude whether practice effects should indeed be considered a novel biomarker of cognitive performance in MS. Ideally, frequent SDMT testing, in an acceptable timeframe, is required to reach a plateau and quantify practice effects on an individual basis, which is difficult to achieve in a clinical setting [5, 6]. Therefore, the development of digital monitoring applications enables further investigation of the practice effect, using a smartphone SDMT (sSDMT) [18, 19, 20, 21, 16]. This sSDMT enabled the real-time and real-world assessment of cognition, possibly increasing sensitivity to short-term fluctuations and reducing time-related variability in processing speed testing. The high testing frequency makes this test ideal for exploring the practice effect.

Therefore, this study aimed to evaluate individual-level practice effects on the sSDMT as a direct proxy of cognitive performance in pwMS. In line with prior studies, we hypothesized that people with more advanced disease show limited practice effects. We tested our hypothesis by evaluating the clinical validity of practice effects by examining 1) the relationship of sSDMT practice effects to cognition, 2) examining the relations with disability, patient-reported cognitive complaints, and brain MRI measures, and 3) by examining the predictive value of practice effects to five-year change in disability and cognition in pwMS.

## Methods

### Participants

Participants of the APPS-MS cohort, a prospective observational cohort study with a one-year observational period followed by a second evaluation at a 5-year follow-up, were included [19, 20, 11]. Eligibility required age 18—65 years at baseline, use of a smartphone, no visual or upper-extremity deficits affecting smartphone use, and no mood, sleep, or concomitant neurologic disorders. PwMS had to be diagnosed with MS according to the 2017 McDonald criteria [22], and were excluded if baseline Expanded Disability Status Scale (EDSS) was ≥ 7.5 or disease modifying therapy switched ≤ 2 months prior to inclusion.

**Fig. 1:**
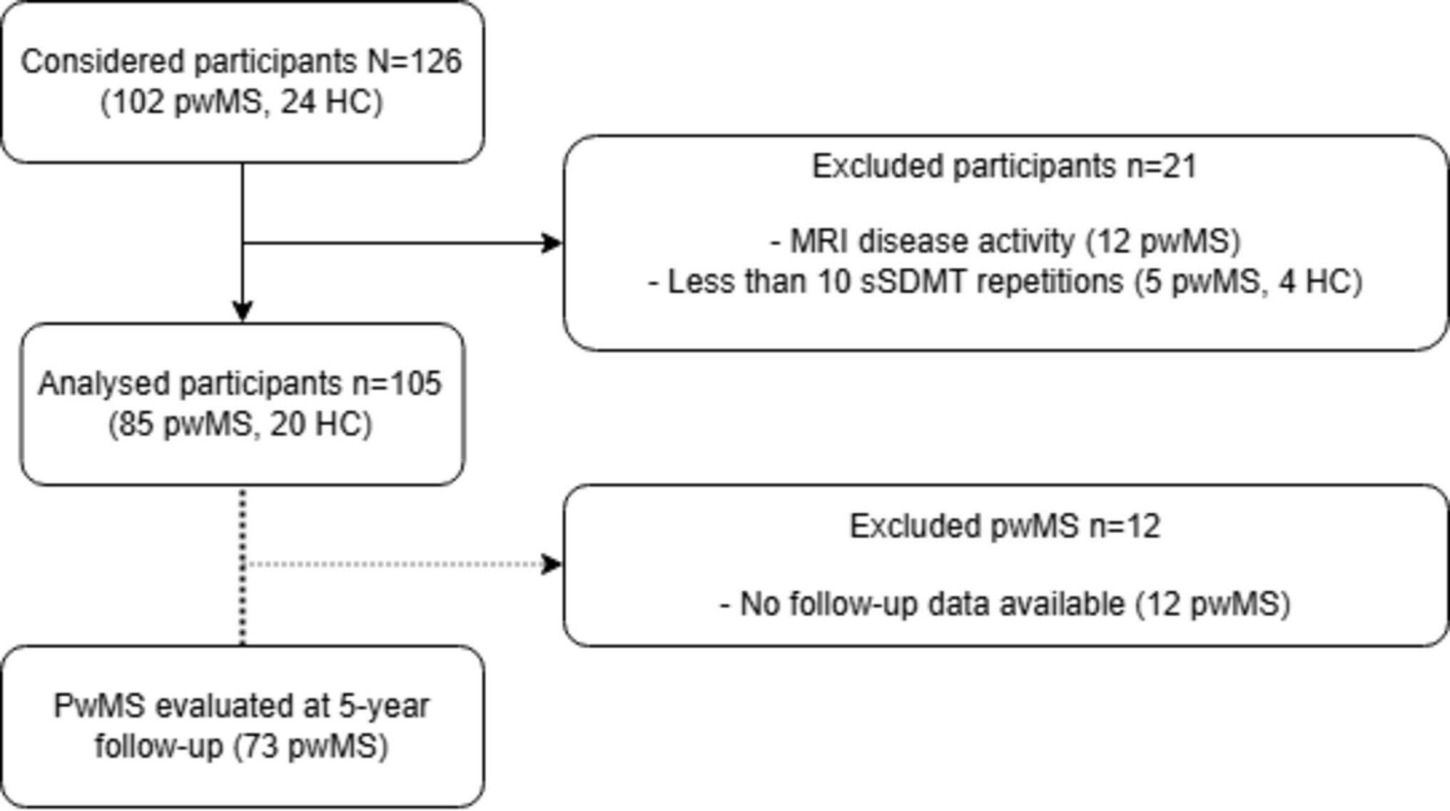
Flowchart of participant inclusion. A part of the considered participants was excluded for reasons listed above. Of the analyzed participants, a part of the participants with MS were re-evaluated after 5 years and were included in the longitudinal analyses

For these analyses, only participants with more than 10 sSDMT test results were considered. To ensure disease stability during the practice period, participants with baseline enhancing lesions or new/enhancing lesions at three months were excluded [23]. Participants lacking a three-month scan were assumed to have no new inflammatory activity (*n* = 2). For an overview of participant inclusion, see Fig. 1.

### Standard protocol approvals, registrations, and patient consents

Participants gave written informed consent. Ethical approval for this study was obtained by the Ethics Committee of the Amsterdam UMC (METC n.2017.576). This study was registered in the Dutch national trial register (NL-OMON27724).

### Clinical evaluation

At baseline and 5-year follow-up, pwMS underwent evaluation with EDSS, Timed 25-Foot Walk Test (T25FW), 9-Hole Peg Test (HPT), and cognitive evaluation. Cognitive testing included an oral Symbol Digit Modalities Test (SDMT), Dutch Verbal Learning Memory Test (Dutch translation of the CVLT-II: VLGT) [24, 25], and Brief Visuospatial Memory Test-Revised (BVMT-R) [26]. Per BICAMS recommendation, the total SDMT, BVMT-R direct recall, and VLGT direct recall scores were analyzed [27]. Education (Verhage scale) was transformed to years of education for analyses [28]. Patient-reported outcomes included the Modified Fatigue Impact Scale [29] (MFIS; cognitive functioning subscale) and Checklist Individual Strength [30] (CIS; concentration problems subscale). MRI acquisition and processing are described in Supplementary Methods S1. Extracted variables include the normalized total brain volume (nTBV), cortex volume (nCortexV), thalamic volume (nThalV), and total lesion volume (LV).

### Digital evaluation

**Fig. 2:**
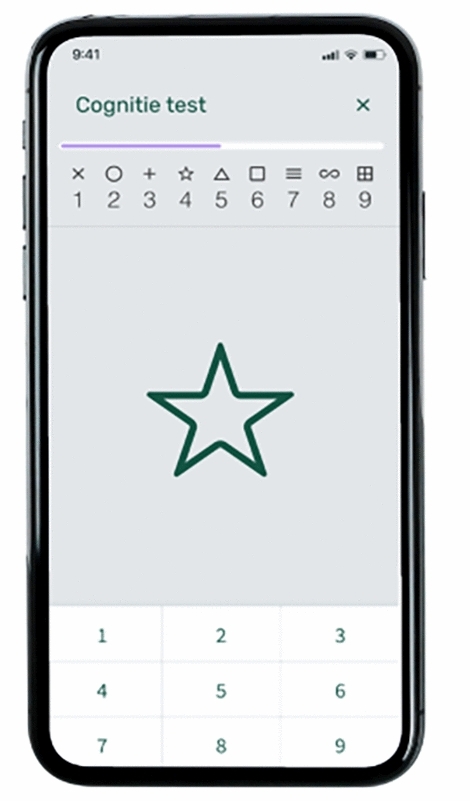
Screenshot of the MS sherpa smartphone SDMT cognition test (screenshot of the Dutch translation). Depicted are the nine symbols (top) that have to be matched using the numbers (below) by selecting one of the numbers in 90 s. The MS Sherpa sSDMT randomly resamples symbols each time a test is performed

Participants used the sSDMT of the MS Sherpa® application (Sherpa, Nijmegen, The Netherlands) [21] daily, without a fixed time of day, for the first five weeks and once every week thereafter up to one year after inclusion. The digital sSDMT differs from the SDMT in that: number-figure matches are selected on a smartphone screen by the participant, the ten practice items preceding the test are not mandatory, the sSDMT randomizes symbols at every administration to prevent sequence memorization, the symbols used are distinct from those in the oral SDMT, and there is no maximum to the number of matches. An example of the test is displayed in Fig. 2. This version of the sSDMT has been validated previously in HC and pwMS [19–21].

### Analyses of digital tests

To quantify the per-individual early-phase sSDMT practice effect in a set time-window, we filtered for the first 40 repetitions. Practice characteristics were quantified using a two-part piecewise linear regression (before and after reaching a practice plateau) fitted to the sSDMT score by test repetition, as was used in a previous study [31]. Using this method, the practice plateau was defined as the repetition at which the initial positive slope of sSDMT performance significantly decreased toward zero, suggesting no further systematic improvement across subsequent repetitions. We selected this approach for its clinically interpretable plateau and because the extracted practice characteristics substantially overlapped with those from more flexible (logarithmic and inverse exponential), though less interpretable methods (Supplement Figures S1–3). Namely, the extracted variables from these more flexible methods all correlated highly with their corresponding variables of the two-part linear regression (baseline sSDMT with the start values of the logarithmic *r* = 0.968 and inverse exponential *r* = 0.949; % increase with increase statistic of the logarithmic *r* = 0.780 and inverse exponential *r* = 0.656 functions; and breakpoint with the breakpoint statistic of the inverse exponential *r* = −0.710), for details see Supplement Figure S3. Moreover, all models showed comparable associations with disability, with consistent negative associations for baseline and plateau performance estimates, whereas learning-rate parameters and breakpoint metrics were not significantly associated with disability (Supplemental Table S1).

**Fig. 3:**
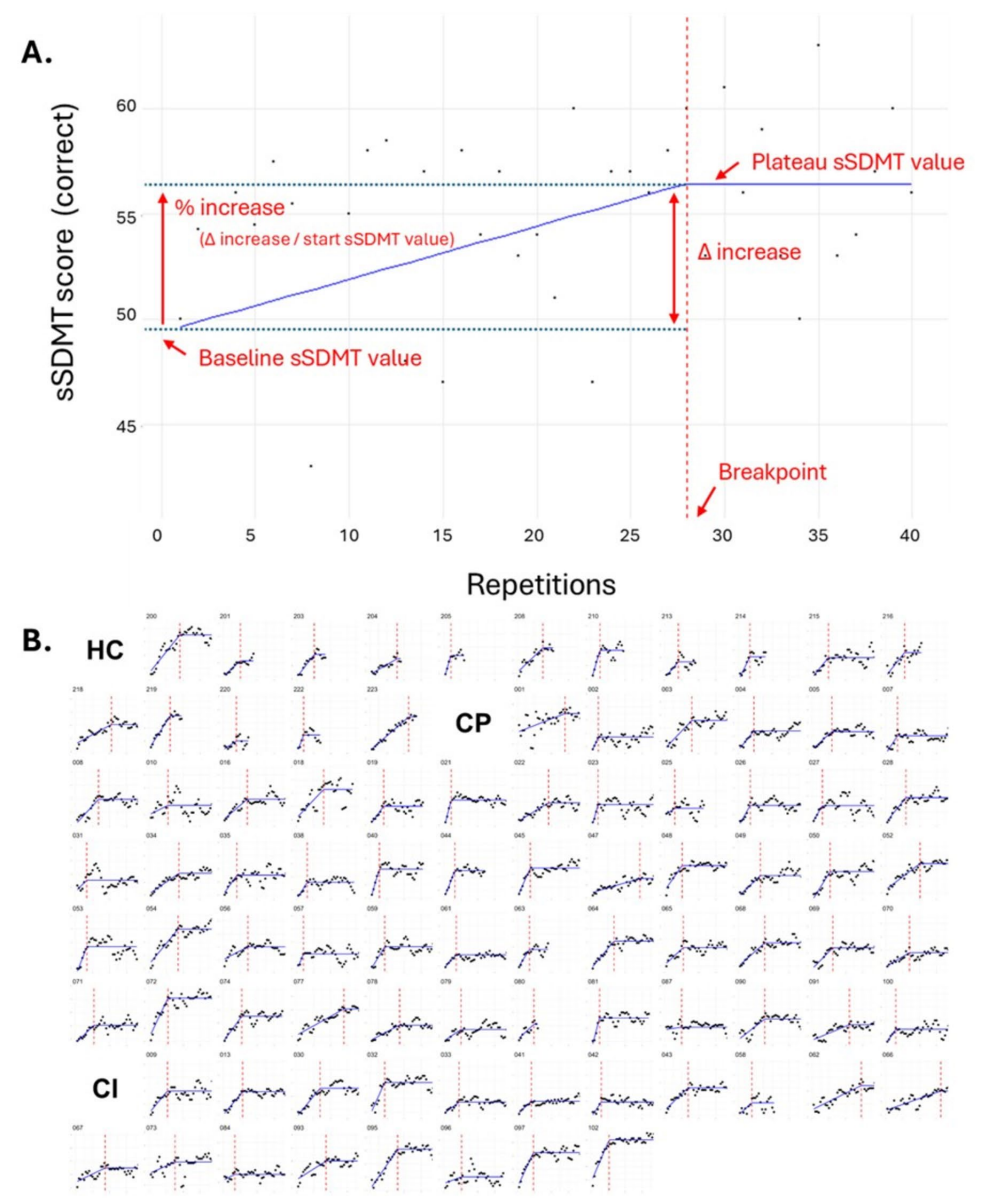
Depiction of variables extracted (**A**) from the breakpoint analysis on the individual curves (**B**). A plateau was determined on an individual-participant level using a two-part piecewise linear regression fitted to the scores on the sSDMT. The extracted variables (**A**) are the baseline sSDMT level, the plateau sSDMT level, the amount of repetitions until the breakpoint is reached, delta increase (Δ-increase), and the percentage increase (Δ-increase/baseline sSDMT level; %-increase). The individual participant curves (**B**) are stratified for healthy controls (HC), cognitively preserved (CP), and cognitively impaired (CI) are also shown

The extracted variables of this analysis are depicted in Fig. 3 and consist of the following: *baseline sSDMT* = y-intercept (baseline) from the pre-plateau linear regression fit; *plateau breakpoint* = number of sSDMT repetitions completed until the start of the plateau; *plateau sSDMT* = predicted sSDMT value from the plateau-phase linear regression fit; *Δ-increase* = the absolute increase between the baseline and plateau sSDMT; *%-increase* = the relative increase calculated as the Δ-increase divided by the baseline sSDMT level. As practice effects depend on testing interval [8], the breakpoint day (*i.e.,* a measure of non-adherence) was quantified to correct for in following analyses.

To evaluate differences between CI and cognitively preserved (CP) pwMS, the cognitive performance was defined using baseline sSDMT *z*-scores relative to the HC means, corrected for age, sex, and education. The sSDMT was used due to the absence of SDMT reference data in this cohort. *Z*-scores of* z* < −1.67 were considered CI, and *z* ≥ −1.67 as cognitively preserved (CP).

### Statistical analysis

Statistical analysis was performed using R v4.3.2 (R Foundation for Statistical Computing, Vienna, Austria). The distribution of the data was checked using histograms and Q–Q plots. Normally distributed data were presented as means with standard deviation (SD) and non-normally distributed data as medians with interquartile ranges (IQR). Log-transformation (applied to: breakpoint, MFIS subscale cognition, and CIS subscale concentration) was applied prior to statistical analysis. Categorical data are presented in percentages. All *p* values were two-sided with a value of *p* < 0.05 considered statistically significant. Cases with missing data were excluded per analysis and no systematic differences were found between complete cases and cases with missing data, see Supplementary Analyses S1.

First, baseline variables were compared between HC/CI/CP using an ANCOVA with Tukey’s HSD-adjusted post hoc comparisons, adjusted for age, sex, and years of education. Age and education were compared using an ANOVA. Thereafter, because sSDMT is a typing test, preliminary checks were performed to assess the impact of HPT on the CI/CP classification. For details, see Supplemental Analysis S2.

#### Determinants of observed practice effects

To evaluate confounding variables, we analyzed the relationships between sSDMT practice effects and age, sex, education, and days until reaching a plateau using Pearson’s correlations in pwMS. Similarly, to evaluate relations among the practice effect outcomes, we analyzed the relations between the extracted practice effect outcomes using partial correlations corrected for age, sex, education, and day at which the breakpoint was reached.

#### Practice effect differences between cognitive groups

To investigate whether individual practice effects differed between HC, CI, and CP, the extracted outcomes were compared using an ANCOVA with Tukey’s HSD-adjusted post hoc comparisons, adjusted for age, sex, education, and days until reaching a plateau. Thereafter, we evaluated whether the pwMS non-learners were more disabled than pwMS learners. We evaluated this using linear regression of clinical and MRI outcomes, adjusted for age and sex.

#### Effect of plateau-based cognitive impairment in pwMS

We then examined the impact of using plateau instead of baseline sSDMT to classify CI/CP. Similar to baseline classification, pwMS were classified relative to HCs using plateau sSDMT values. In each participant in whom no plateau was identified, the baseline sSDMT was used instead. To assess agreement between baseline versus the plateau CI classification, we used a chi-square test. Similar analyses were performed excluding participants without an identifiable plateau (see Supplemental Figure S5).

#### Associations to clinical variables, patient-reported outcomes, and brain volumes

To examine the relation between the practice characteristics and clinical performance at baseline in the pwMS, as part of the evaluation of the clinical validity of practice effects, we performed linear regression using the practice characteristics as independent variable, adjusted for age, sex, education, and days until reaching a plateau. Bonferroni multiple testing correction was applied. Similar regression was performed in relation to patient-reported outcomes and MRI brain volumes.

#### Associations to disease worsening and cognitive decline

Then, to evaluate the predictive value of early-phase practice effects, we examined whether associations existed in relation to future outcomes in MS. To achieve this, we performed linear regression predicting follow-up outcomes with baseline clinical performance, adjusted for age, sex, education, and days until reaching a plateau. Explained variance of the practice effects outcomes was calculated. Bonferroni multiple testing correction was applied. Follow-up clinical and cognitive outcomes were compared to baseline using paired t-tests and Wilcoxon signed-rank tests.

## Results

### Participant demographics and digital testing

85/102 pwMS and 20/24 HC with sufficient repetitions and no inflammatory disease activity on MRI were considered for analyses, see Fig. 1. For demographic characteristics of the cohort, see Table 1**.** Based on the baseline sSDMT value, 19/85 pwMS were classified CI. The mean age and years of education were comparable between the HC/CI/CP groups (*p* > 0.05). The CI group showed more advanced disease compared to the CP group: the CI had a longer disease duration, lower SDMT, higher HPT, lower nTBV, lower nThalV, lower nCortexV, and higher LV compared to CP (all adj.*p* < 0.05). EDSS and T25FW were not different between CP and CI pwMS (adj.*p* > 0.05). The pwMS scored higher on the patient-reported outcomes compared to the HC (adj.*p* < 0.05) and CI scored higher on the MFIS but lower on the CIS (adj.*p* < 0.05) compared to the CP.

**Table 1:** Participant characteristics

		HC (*n* = 20)	CP (*n* = 66)	CI (*n* = 19)	*adj.p*	
Age, years		46.0 (14.0)	46.5 (10.2)	51.6 (7.85)	*0.160*	
Female sex (*n*, %)		11 (55.0%)	52 (78.8%)	8 (42.1%)		
Education, years		16.0 [15.8, 18.0]	16.0 [15.0, 16.0]	16.0 [15.0, 16.0]	*0.375*	
MS subtype		–	RRMS: 39 (59.1%), SPMS: 17 (25.8%), PPMS: 10 (15.2%)	RRMS: 6 (31.6%), SPMS: 12 (63.2%), PPMS: 1 (5.3%)		
Disease duration, years		–	5.12 [2.81, 11.0]	12.1 [6.81, 17.4]	***0.013***	†
DMT line (*n*, %)		–	First line: 32 (48.5%), Second line: 7 (10.6%)	First line: 8 (42.1%), Second line: 3 (15.8%)	*0.798*	
EDSS		–	3.25 [2.50, 4.00]	4.00 [3.50, 4.25]	*0.140*	
SDMT		–	56.2 (9.07)	46.7 (9.81)	** < *0.001***	†
VLGT		–	56.5 (9.00)	44.1 (10.0)	** < *0.001***	†
BVMT-R		–	26.7 (5.18)	21.5 (7.11)	** < *0.001***	†
HPT, s		–	19.7 [18.2, 22.3]	23.0 [20.9, 30.7]	** < *0.001***	†
T25FW, m		–	4.61 [4.00, 5.45]	5.28 [4.44, 6.20]	*0.195*	
MFIS cognitive subscale		4.00 [0, 9.50]	16.0 [10.0, 23.0]	19.0 [12.0, 26.8]	** < *0.001***	‡
CIS concentration subscale		7.00 [5.00, 12.0]	19.0 [11.0, 23.0]	14.5 [8.50, 23.0]	** < *0.001***	‡
nTBV, L/sbTIV		0.706 (0.0187)	0.697 (0.0205)	0.671 (0.0194)	***0.009***	†
nThalV, mL/sbTIV		8.31 (0.413)	7.98 (0.574)	7.07 (0.533)	** < *0.001***	*
nCortexV, L/sbTIV		0.322 (0.0199)	0.317 (0.0128)	0.307 (0.0142)	***0.034***	†
Lesion volume, mL		–	3.88 [1.92, 8.77]	14.1 [6.40, 22.0]	** < *0.001***	†

Bold indicates statistical significance

Demographic characteristics of the included participants. Results are stratified into healthy controls (HC), cognitively preserved (CP), and cognitively impaired (CI). Numbers are either mean with a standard deviation or median with an IQR. Disease duration is from date of diagnosis of MS. Baseline characteristics are compared using an ANCOVA with Tukey’s HSD-adjusted post hoc comparisons, adjusted for age, sex, and years of education. An ANOVA was performed for age and education

*BVMT-R* Brief Visuospatial Memory Test – Revised, *CIS* Checklist Individual Strength, *DMT* disease-modifying therapy, *EDSS* Expanded Disability Status Scale, *HPT* 9-Hole Peg Test, *MFIS* Modified Fatigue Impact Scale, *MS* multiple sclerosis, *nCortexV* normalized cortical volume, *nTBV* normalized total brain volume, *nThalV* normalized thalamic volume, *sbTIV* segmentation-based Estimate of Total Intracranial volume, *SDMT* Symbol Digit Modalities Test, *T25FW* Timed 25-Foot Walk, *VLGT* Verbal Learning and Memory Test (Dutch version of the CVLT-II)

Post hoc comparison: * CI, CP, and HC differed from each other. † CI differed from CP. ‡ CI and CP differed from HC

The HC performed a median of 18.5 repetitions [IQR: 16.8—21.0]. The pwMS performed more tests (CP: 57.5 [IQR: 34.2—71.5]; CI: 62.0 [IQR: 50.0—136.0]). A plateau could be identified in 16/20 HC, 54/66 CP, and 19/19 CI with a *R*^2^ = 0.291. For the individual practice characteristics see Table 2.

**Table 2:** Individual practice effect parameters for HC, CI, and CP

Outcome	Group			*F* value	*adj. p*	
	HC	CP	CI			
# reached plateau	16	54	19			
Breakpoint	10.38 (7.71—15.22)	10.74 (7—14.65)	10.72 (8.78—15.72)	1.11	*0.335*	
Baseline sSDMT value	48.05 ± 7.28	46.06 ± 5.59	32.78 ± 6.48	***55.03***	** < *0.001***	*†*
Plateau sSDMT value	55.8 ± 7.54	53.11 ± 6.15	40.03 ± 8.67	***37.52***	** < *0.001***	*†*
∆-increase	7.75 ± 2.76	7.05 ± 2.44	7.25 ± 3.71	0.38	*0.684*	
%-increase	16.56 ± 6.42	15.52 ± 5.82	22.04 ± 10.71	***6.05***	***0.004***	*†*

Bold indicates statistical significance

Mean ± SD or Median (IQR1–IQR3)

Results of group comparison between healthy controls (HC), cognitively preserved (CP), and cognitively impaired (CI) of participants with an identifiable plateau (# reached plateau). Comparison was performed using an ANCOVA adjusted for age, sex, education, and days until plateau was reached. A Tukey’s HSD-adjusted post hoc comparison was performed

Post hoc results are indicated using: † wherein CI differed from CP

### Determinants of observed practice effects

In pwMS, older age was associated with a later breakpoint (*r* = 0.340 (95%-CI: 0.142,0.512), *p* = 0.001), lower baseline sSDMT (*r* = −0.522 (95%CI: −0.658,−0.352), *p* < 0.001), lower plateau sSDMT (*r* = −0.463 (95%CI: −0.612,−0.282), *p* < 0.001), and higher %-increase (*r* = 0.295 (95%CI: 0.092,0.474), *p* = 0.005). Female sex was related to a higher plateau sSDMT (*r* = 0.211 (95%CI: 0.002, 0.401), *p* = 0.048). Education was related to a higher baseline sSDMT (*r* = 0.323 (95%CI: 0.123, 0.498), *p* = 0.002) and higher plateau sSDMT (*r* = 0.317 (95%CI: 0.116, 0.492), *p* = 0.003). The breakpoint repetition weakly related to the breakpoint day (*r* = 0.258 (95%CI: 0.052, 0.442), *p* = 0.015).

We evaluated associations among cognition and practice effect characteristics and found baseline sSDMT was highly related to the plateau sSDMT (*ρ* = 0.930 (95%CI: 0.894, 0.954), *p* < 0.001) and lower %-increase (*ρ* = −0.266 (95%CI:−0.452,−0.057), *p* = 0.013). The plateau sSDMT related to higher ∆-increase (*ρ* = 0.472 (95%CI: 0.289, 0.622), *p* < 0.001). The ∆-increase related to %-increase (*ρ* = 0.900 (95%CI: 0.850, 0.934), *p* < 0.001) and a later breakpoint (*ρ* = 0.224 (95%CI: 0.013, 0.416), *p* = 0.038).

### Practice effect differences between cognitive groups

**Fig. 4:**
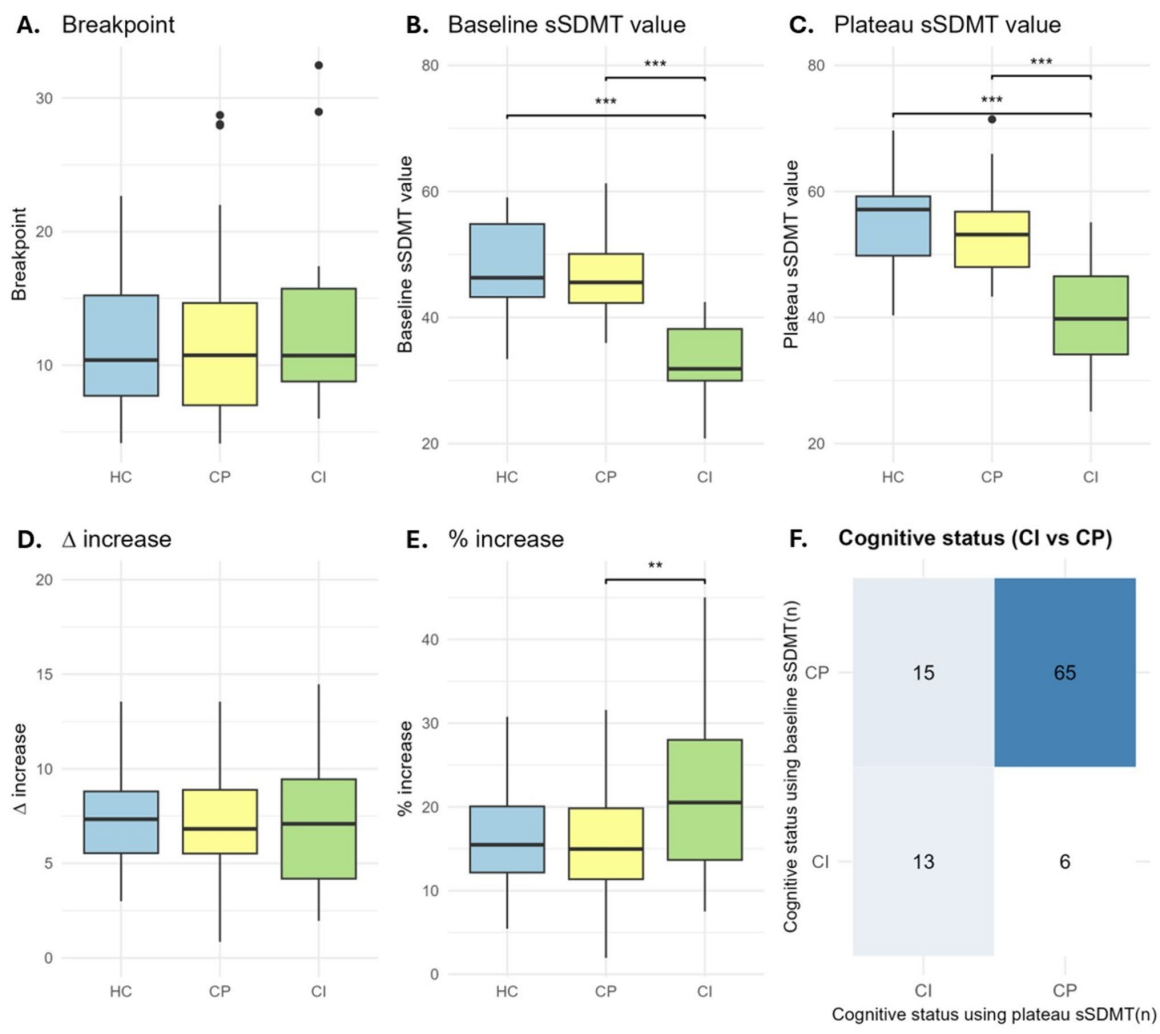
Extracted variables from the individual practice effect curves. Depicted are boxplots (median and IQR) extracted variables of the individual-level practice effect curves stratified into healthy controls (HC), cognitively preserved (CP), and cognitively impaired (CI) based on the baseline sSDMT results. Depicted are (**A**) the amount of repetitions until the breakpoint is reached, (**B**) baseline sSDMT level, (**C**) plateau sSDMT level, (**D**) absolute delta increase (Δ-increase), and (**E**) the percentage increase (Δ-increase/baseline sSDMT level). Additionally, the classification of CI and CP is shown using either the baseline sSDMT or the plateau sSDMT relative to the start or plateau sSDMT of the HCs (**F**). The practice effect outcomes are compared using an ANCOVA with Tukey’s HSD-adjusted post hoc comparisons, adjusted for age, sex, education, and days until reaching a plateau. Only statistically significant results are depicted wherein *adj.*p* < 0.05, **adj.*p* < 0.01, and ***adj.*p* < 0.001

We compared practice outcomes across HC/CP/CI groups, see Fig. 4A–E. First, in line with the definition of the groups, we found baseline sSDMT was the lowest in CI compared to HC (adj. mean difference = 13.50 (95%CI: 9.29, 17.71), adj.*p* < 0.001) and CP (adj. mean difference = 11.42 (95%CI: 7.97,14.88, adj.*p* < 0.001). There was no difference between CP and HC (adj. mean difference = 2.07 (95%CI: −1.54, 5.69), adj.*p* = 0.361). Similarly, the plateau sSDMT value was lowest in CI (CI vs CP: adj. mean difference = 11.00 (95%CI: 6.80, 15.20), adj.*p* < 0.001; CI vs HC: adj. mean difference = 13.94 (95%CI: 8.82, 19.06), adj.*p* < 0.001), and not lower in CP compared to HC (adj. mean difference = 2.94 (95%CI: −1.46,7.33), adj.*p* = 0.253). Post hoc results indicated that CI had a higher %-increase compared to the CP (adj. mean difference = 6.26 (95%CI: 1.48, 11.05), adj.*p* = 0.007), whereas the ∆-increase (adj. mean difference = 0.42 (95%CI:−1.48, 2.33), adj.*p* = 0.684) did not differ. The HC/CI/CP groups reached a plateau at a similar breakpoint repetition (*F* = 1.11, adj.*p* = 0.335) and the plateau day also did not differ between groups: HC = 21 days [IQR: 15–35], CP = 19 days [IQR: 14–38]; CI = 28 days [IQR: 16–53], (*F* = 2.52, adj.*p* = 0.087).

### Characteristics of participants without a practice effect

3 HC and 8 pwMS performed at least 15 repetitions but no plateau could be detected, see Supplement Figure S4. These pwMS were all CP, were not distinguishable from pwMS with a plateau (age, disease duration, EDSS, T25FW, HPT, MFIS cognition subscale, CIS concentration subscale, nTBV, nCortexV, nThalV, LV; all *p* > 0.05, estimates not presented), and their follow-up outcomes (EDSS, HPT, TWT, BVMT-R, VLGT, SDMT) were not statistically different from baseline (all *p* > 0.05, values not presented).

### Effect of plateau-based cognitive impairment in pwMS

Then, we examined whether plateau versus baseline SDMT altered CI classification, see Fig. 3F. We found that baseline sSDMT classified 19/85 (22.4%) pwMS CI (*z* = −0.679 ± 1.31), whereas this was 23/85 (27.1%) using the plateau sSDMT (*z* = −0.664 ± 1.53). Of the pwMS who were regarded CI using either classification, there was an overlap of 45% (*X*^2^ = 16.31, *p* < 0.001). With the plateau sSDMT CI/CP classification, the differences between CP/CI/HC indicated that the ∆-increase was now higher for the CI compared to the HC (adj. mean difference = 2.37 (95%CI: 0.13, 4.61), adj.*p* = 0.036) and lower compared to CP (adj. mean difference = −2.25 (95%CI: −4.06, −0.435), adj.*p* = 0.011), see Supplement Figure S5. Post hoc analyses indicated that the shift in CI/CP classification for either the baseline or the plateau sSDMT persisted after excluding non-plateauing participants Supplemental Figure S6.

### Associations to clinical variables, patient-reported outcomes, and brain volumes

Furthermore, we evaluated the clinical validity of the practice effect characteristics as proxy of cognitive performance and markers of disability and found that there were no associations between the practice characteristics (repetition breakpoint, ∆-increase, and %-increase) and the clinical or patient-reported outcomes, see Table 3. Moreover, our analyses revealed that relations of the clinical variables to the plateau sSDMT value were stronger than to the baseline sSDMT (plateau vs baseline sSDMT; HPT: std.*β* = −0.539 vs −0.471, T25FW: −0.339 vs −0.332, SDMT: 0.545 vs 0.461, VLGT: 0.500 vs 0.396).

**Table 3:** Relation of practice characteristics to clinical outcomes and MRI measures

		Breakpoint	Baseline sSDMT	Plateau sSDMT	∆-increase	%-increase
Clinical variables						
EDSS		0.027 (−0.220—0.275), *p* = 0.826	−0.275 (−0.478—−0.072), *p* = 0.009	−0.223 (−0.442—−0.003), *p* = 0.047	0.098 (−0.179—0.375), *p* = 0.482	0.186 (−0.076—0.447), *p* = 0.161
HPT		−0.119 (−0.349—0.111), *p* = 0.306	−**0.471 (**−**0.635—**−0.307), *p* < 0.001*	−**0.539 (**−**0.704—**−0.373), *p* < 0.001*	−0.334 (−0.581—−0.087), *p* = 0.009	−0.169 (−0.413—0.076), *p* = 0.174
T25FW		−0.141 (−0.366—0.084), *p* = 0.215	−**0.332 (**−**0.505—**−0.159), *p* < 0.001*	−**0.339 (**−**0.529—**−0.148), *p* = 0.001*	−0.106 (−0.35—0.137), *p* = 0.387	0.007 (−0.21—0.224), *p* = 0.951
SDMT		0.003 (−0.248—0.254), *p* = 0.980	0.461 (0.275—0.646), *p* < 0.001*	0.545 (0.359—0.732), *p* < 0.001*	0.388 (0.122—0.653), *p* = 0.005	0.106 (−0.162—0.374), *p* = 0.433
BVMT-R		0.059 (−0.199—0.316), *p* = 0.650	0.319 (0.110—0.527), *p* = 0.003	0.333 (0.112—0.554), *p* = 0.004	0.126 (−0.162—0.413), *p* = 0.386	−0.087 (−0.361—0.188), *p* = 0.531
VLGT		0.058 (−0.199—0.314), *p* = 0.656	0.396 (0.197—0.596), *p* < 0.001*	0.500 (0.300—0.700), *p* < 0.001*	0.430 (0.162—0.698), *p* = 0.002	0.181 (−0.09—0.452), *p* = 0.188
Patient-reported outcomes						
MFIS cognitive subscale		−0.102 (−0.324—0.121), *p* = 0.365	−0.122 (−0.318—0.073), *p* = 0.216	−0.173 (−0.375—0.029), *p* = 0.092	−0.192 (−0.443—0.058), *p* = 0.130	−0.099 (−0.343—0.146), *p* = 0.423
CIS concentration subscale		−0.106 (−0.326—0.113), *p* = 0.337	−0.137 (−0.329—0.055), *p* = 0.159	−0.193 (−0.391—0.004), *p* = 0.055	−0.215 (−0.461—0.03), *p* = 0.084	−0.090 (−0.331—0.151), *p* = 0.459
MRI variables						
nTBV		−0.217 (−0.495—0.062), *p* = 0.125	0.169 (−0.039—0.377), *p* = 0.110	0.169 (−0.05—0.387), *p* = 0.127	0.043 (−0.256—0.342), *p* = 0.775	−0.067 (−0.36—0.227), *p* = 0.651
nThalV		−0.095 (−0.363—0.174), *p* = 0.485	0.202 (0.007—0.397), *p* = 0.043	0.213 (0.008—0.417), *p* = 0.042	0.084 (−0.199—0.368), *p* = 0.554	0.011 (−0.269—0.29), *p* = 0.939
nCortexV		−0.030 (−0.363—0.304), *p* = 0.860	0.065 (−0.184—0.313), *p* = 0.606	0.126 (−0.133—0.386), *p* = 0.334	0.214 (−0.133—0.561), *p* = 0.223	0.151 (−0.193—0.494), *p* = 0.384
LV		0.068 (−0.187—0.324), *p* = 0.594	−0.256 (−0.436—−0.076), *p* = 0.006	−**0.306 (**−**0.491—**−0.120), *p* = 0.002*	−0.223 (−0.487—0.041), *p* = 0.096	−0.117 (−0.38—0.147), *p* = 0.380

std.β (95%CI), *p* value

Relations between practice effect characteristics, clinical performance and MRI measures at baseline for people with MS. Extracted outcomes are the baseline sSDMT level, the plateau sSDMT level, the amount of repetitions until the breakpoint is reached (Breakpoint), delta increase (Δ-increase), and the percentage increase (%-increase = Δ-increase/baseline sSDMT level). Analyses were linear regressions, adjusted for age, sex, education, and days until reaching a plateau, corrected for multiple testing using Bonferroni. Presented *p* values are uncorrected

*CIS* Checklist Individual Strength, *EDSS* Expanded Disability Status Scale, *HPT* 9-Hole Peg Test, *MFIS* Modified Fatigue Impact Scale, *nCortexV* normalized cortical volume, *nTBV* normalized total brain volume, *nThalV* normalized thalamic volume, *SDMT* Symbol Digit Modalities Test, *T25FW* Timed 25-Foot Walk

^*^ and bold indicates significance after Bonferroni multiple testing correction

Among the MRI variables only LV was associated to the plateau sSDMT (std.*β* =  − 0.306 (95%CI: − 0.491, − 0.120), *p* = 0.002), but the relation between LV and the baseline sSDMT (std.*β* =  − 0.256 (95%CI: − 0.436, − 0.076), *p* = 0.006) was not significant after correction for multiple testing.

### Disease worsening and cognitive decline and associations with practice effect outcomes

73/85 pwMS were re-evaluated at a follow-up visit after a mean of 5.39 ± 0.38 years. The EDSS increased from 3.50 [IQR: 3.00–4.00] to 4.00 [IQR: 2.50–6.00] (*W* = −0.50, *p* = 0.004), as did the T25FW from 4.78s [IQR: 4.21–5.90] to 5.11s [IQR: 4.41–6.48] (*W* = −0.80, *p* < 0.001), while the HPT, BVMT-R and VLGT remained stable (at baseline vs follow-up: HPT: 20.6 [IQR: 19.0–23.9] vs 20.4 [IQR: 18.8–24.7] (*W* = 0.00, *p* = 1.00), BVMT-R: 25.4 ± 6.07 vs 24.8 ± 6.73 (t(*50*) = 0.82 (95%CI: −0.792, 1.89), *p* = 0.415), VLGT: 53.2 ± 10.6 vs 53.7 ± 12.3 (t(*51*) = −1.17, (95%CI: −3.032, 0.801), *p* = 0.248)). Interestingly, an increase was observed in the oral SDMT from 53.9 ± 10.3 to 56.6 ± 13.6 (t(*54*) = −2.69, (95%CI: −4.80,−0.58), *p* = 0.014).

To assess whether practice effects explained longitudinal clinical change, extracted measures were added to linear regression models, see Table 4. No practice outcome contributed more than 10% to explaining variance in the baseline-follow-up models, except for baseline and plateau sSDMT values, that explained change in the SDMT (baseline sSDMT explained 14.04% (std.*β* = 0.528 (95%CI: 0.317,0.699), *p* < 0.001, cor.*p* < 0.001), plateau sSDMT explained 13.84% (std.*β* = 0.523 (95%CI: 0.303,0.675), *p* < 0.001, cor.*p* < 0.001)). The practice outcomes that best explained change in a clinical outcome were the breakpoint (7.61% (std.*β* = 0.232 (95%CI: 0.039,0.413), *p* = 0.019, cor.*p* = 0.558)), baseline SDMT (7.46% (std.*β* = −0.392 (95%CI: −0.699,−0.063), *p* = 0.020, cor.*p* = 0.597)), and %-increase (5.62% (std.*β* = 0.199 (95%CI: 0.003,0.421), *p* = 0.047, cor.*p* = 1.000)) in explaining the longitudinal HPT, but these were not significant after multiple testing comparisons.

**Table 4:** Linear regression to longitudinal outcomes, corrected for baseline outcome, age, sex, education, and days until reaching a plateau

			% of variance explained	std.*β*	95%CI	*p* value	Bonferroni-corrected *p* value
EDSS	BL – 5Y FU	Breakpoint	4.42%	−0.164	(−0.337,0.019)	0.079	
		Baseline sSDMT	0.35%	−0.058	(−0.297,0.181)	0.630	
		Plateau sSDMT	0.22%	−0.042	(−0.259,0.177)	0.707	
		∆ increase	0.01%	0.007	(−0.175,0.190)	0.936	
		% increase	0.03%	0.014	(−0.186,0.214)	0.888	
HPT	BL – 5Y FU	Breakpoint	7.61%	0.232	(0.039,0.413)	0.019	0.558
		Baseline sSDMT	7.46%	−0.392	(−0.699,−0.063)	0.020	0.597
		Plateau sSDMT	5.01%	−0.302	(−0.585,0.015)	0.062	
		∆ increase	0.08%	0.023	(−0.180,0.227)	0.818	
		% increase	5.62%	0.199	(0.003,0.421)	0.047	1.000
T25FW	BL – 5Y FU	Breakpoint	0.07%	−0.023	(−0.191,0.146)	0.792	
		Baseline sSDMT	1.3%	−0.151	(−0.385,0.098)	0.239	
		Plateau sSDMT	1.17%	−0.132	(−0.338,0.095)	0.264	
		∆ increase	0.11%	−0.029	(−0.194,0.137)	0.732	
		% increase	0%	0.006	(−0.177,0.190)	0.946	
SDMT	BL – 5Y FU	Breakpoint	0.36%	−0.054	(−0.218,0.113)	0.527	
		Baseline sSDMT	14.04%	0.528	(0.317,0.699)	0.000	0.000
		Plateau sSDMT	13.84%	0.523	(0.303,0.675)	0.000	0.000
		∆ increase	0.89%	0.090	(−0.089,0.268)	0.319	
		% increase	0.2%	−0.041	(−0.227,0.141)	0.642	
BVMT-R	BL – 5Y FU	Breakpoint	0.12%	0.030	(−0.173,0.230)	0.779	
		Baseline sSDMT	1.25%	0.120	(−0.143,0.386)	0.360	
		Plateau sSDMT	2%	0.145	(−0.102,0.393)	0.243	
		∆ increase	1.34%	0.095	(−0.105,0.296)	0.343	
		% increase	0.25%	0.042	(−0.174,0.262)	0.686	
VLGT	BL – 5Y FU	Breakpoint	1.66%	−0.116	(−0.282,0.063)	0.210	
		Baseline sSDMT	3.56%	0.234	(−0.010,0.461)	0.061	
		Plateau sSDMT	3.01%	0.213	(−0.030,0.436)	0.086	
		∆ increase	0%	0.004	(−0.191,0.199)	0.967	
		% increase	0.35%	−0.055	(−0.258,0.143)	0.567	

Bold indicates statistical significance

Explanatory value of practice effect outcomes in explaining longitudinal change in clinical outcomes between baseline and 5-year follow-up. Presented results are the % of variance explained by the practice outcome, the standardized β, 95% confidence intervals (CI), and the *p* value of the practice outcome in linear regression models predicting the long-term outcome adjusted for the baseline clinical performance, age, sex, education, and days until reaching a plateau, with addition of the practice outcomes. The corrected *p* value is after applying Bonferroni multiple testing correction

## Discussion

This study aimed to evaluate individual-level practice effects on the sSDMT as a marker of cognitive performance, overall disease severity and long-term disease worsening in pwMS. Our results indicated that practice effect characteristics were associated with cognitive performance. Specifically, higher baseline cognitive performance was related to a higher %-increase and higher sSDMT plateau. However, the practice effect was neither unique to MS patients nor disease severity; the practice effect was similar in pwMS compared to HCs, and we found no associations with disability, patient-reported outcomes, brain measures, and clinical outcomes at follow-up. Additionally, we demonstrated that individual-level practice effects influenced the classification of whether people were considered cognitively impaired, and associations to disease severity altered between the baseline and the plateau sSDMT score.

We found evidence supporting the clinical validity of practice effects as a proxy of cognition, but practice effects were largely unrelated to measures of disease severity or longitudinal clinical outcomes. Nevertheless, our findings contradicted our hypothesis and findings from prior studies, wherein the practice effect was found to be a proxy of cognitive performance as it was more pronounced in pwMS with mild disability [12, 13, 15]. An example is a large-scale study that accessed practice effects on a cognitive processing speed test over four years, but with longer between-visit intervals of up to one year. This study found that worse baseline cognition and the lack of practice effect predicted future cognitive performance in MS [13]. Interestingly, in contrast, our results indicated that a lack of a practice effect (~ 10% of pwMS) did not signify worse cognitive or clinical performance. Moreover, with regards to relations to brain volumes, other studies found reduced short-term PASAT learning to be related to brain atrophy in MS [14], and reduced gray and white mater atrophy in patients with a clinically isolated syndrome [15]. This could be due to difference in practice effects between the PASAT and the SDMT [32]. Nevertheless, evidence on the relation between MRI brain measures and practice effects is not consistent as another study found no relationship between SDMT practice effects and brain atrophy [9].

Several factors could have influenced our findings. First, we included HCs, which allowed us to observe that practice effects were not unique to disease status, highlighting the value of including HC data in this field of research. Next, we only included pwMS with sufficient arm function to operate a smartphone, meaning that pwMS in our cohort showed relatively mild disability and only few classified as cognitively impaired [13, 9, 17]. Thirdly, there were drastic differences in the testing intervals in other studies (monthly or yearly) compared to our study were testing was performed daily [13, 9, 10, 17]. Consequently, in our analyses, improvements in cognitive scores likely related more purely to practice effects, whereas longer intervals introduce ongoing gradual cognitive decline as well. Consequent to this, longer intervals may allow sSDMT practice effect variability to also reflect accumulated decline, increasing the likelihood of correlation with further decline.

Several findings from our study highlight the importance of accounting for practice effects in studies with frequent cognitive processing speed assessments. First, the sSDMT practice effect might have influenced oral SDMT outcomes as a 2.7 points increase was measurable four years after discontinuing smartphone assessments. This increase potentially hinders the detection of cognitive decline on both the sSDMT and the oral SDMT. Future studies can use this knowledge to identify and quantify long-term cognitive decline independent of practice effects, but novel approaches should also be explored, such as identifying temporal intra-trial characteristics that are immune to practice effects [33]. Second, we demonstrated that associations of clinical and brain measures in relation to the plateau sSDMT were slightly stronger than to the baseline sSDMT. Therefore, when the window of testing is not considered, observed variability in sSDMT testing may reflect practice effects rather than MS pathology. Third, we showed that this window of testing also affected the CI/CP classification. Although the baseline sSDMT highly correlated to the plateau sSDMT, the CI/CP classification at baseline differed from the classification after the plateau. Specifically, there was only an overlap of 45% in pwMS considered CI using either classification.

Nevertheless, the plateau sSDMT may also provide additional insights into cognition, given its stronger associations with disease severity than baseline measurements. A possible explanation for this may be a regression to the mean, a statistical phenomenon that reflects the tendency for outlier measurements to regress toward the mean upon retesting [9]. A finding supporting this hypothesis is the counterintuitive observation that CI pwMS practice scores increased more than CP pwMS although CI had overall poorer clinical characteristics at baseline [10, 17].

Several methodological limitations may have reduced the ability to detect associations and should be considered when interpreting the null findings. First, the cohort was relatively mildly affected, resulting in limited variability in clinical characteristics and potentially limiting detectable associations. Moreover, the cohort was relatively small, impacting the statistical power of our analyses. Second, we only explored whole brain and large regional brain volumes. Hypothesis-driven regional analyses may have been more sensitive to potentional relationships. Third, our version of the sSDMT relies on keypad input. We found little confounding effects from upper extremity motor function, yet the sSDMT cannot be interpreted independently of motor function. Future studies should replicate our findings with tests, such as the digital oral SDMT and expand the scope of practice effects, toward additional cognitive domains [34]. Fourth, we focused on the first 40 repetitions, given that the majority of the practice effect occurs in this early phase. However, there is evidence that the practice effect might continue on the long term [17, 16] or that no true plateau exists [8]. Therefore, future studies need to address the prognostic value of late-phase practicing. Finally, we identified various non-linear functions to quantity practice effects that fit the data better. We used a two-part linear regression because of the interpretability of the plateau for future applications. However, we acknowledge that the use of a different model-based method might have resulted in different results, although we found no evidence supporting this in our supplementary analyses using other non-linear functions, as relations among practice effects extracted using different model-based methods were high and similar relations were found to disability.

In conclusion, we found evidence for a relationship between early-phase sSDMT practice effects and cognition, but this relation was not specific to MS, and this effect was unrelated to disease severity and disease worsening at five-year follow-up. It is crucial to consider the combination of practice effects and plateauing of the SDMT when accessing cognitive information processing speed in pwMS, as the practice effect altered the classification of cognitive impairment in pwMS and changed associations of the sSDMT with disability and brain volumes. Moreover, the sSDMT practice effect remained measurable four years after discontinuing smartphone sSDMT testing. Therefore, clinicians and researchers need to be aware of the practice effect when assessing results from high frequency cognitive information processing speed testing in MS trials.

## Conflicts of interest

The author(s) declared the following potential conflicts of interest with respect to the research, authorship, and/or publication of this article: D.J. de Jong declares no conflict of interest.; P.C.G. Molenaar declares no conflict of interest.; V. Verhoef declares no conflict of interest.; L. Novakova has received lecture honoraria from Biogen, Novartis, Teva, Sanofi and Merck, has served on advisory boards for Merck, Janssen, Argenx and Sanofi, and has received unconditional grants from Novartis and Sanofi.; E. Colato receives grant support from MAGNIMS and research support from EIP.; T.A.A. Broeders declares no conflict of interest.; S. Noteboom declares no conflict of interest.; K.H. Lam declares no conflict of interest.; S. Cloosterman is an employee of MS Sherpa.; B. Moraal declares no conflict of interest.; D. Nieuwkamp declares no conflict of interest.; O. Gerlach declares no conflict of interest.; J. Mostert declares has nothing to declare.; E.M.M. Strijbis reports speaker relations with Merck and Novartis (all payments to institution) and served on advisory board for Roche.; M.M. Schoonheim serves on the editorial board of Neurology and Frontiers in Neurology, receives research support from the Dutch MS Research Foundation, Eurostars-EUREKA, ARSEP, Amsterdam Neuroscience, MAGNIMS and ZonMW (Vidi grant, project number 09150172010056) and has served as a consultant for or received research support from Atara Biotherapeutics, Biogen, Celgene/Bristol Meyers Squibb, EIP, Sanofi, MedDay and Merck.; J. Killestein received consulting fees for F. Hoffmann-La Roche, Biogen, Teva, Merck, Novartis and Sanofi/Genzyme (payments to institution); reports speaker relationships with F. Hoffmann-La Roche, Biogen, Teva, Merck, Novartis and Sanofi/Genzyme (payments to institution); adjudication committee of MS clinical trials of Immunic (payments to institution).; T.A. Fuchs received research support from the European Committee for Treatment and Research in Multiple Sclerosis, serves on the editorial board of Frontiers in Neurology, and received consulting fees for Click Therapeutics and Registry Multiple Sclerosis (ReMuS).

## Supplementary Information

Below is the link to the electronic supplementary material.

Supplementary file1 (DOCX 4262 KB)

## Data Availability

Anonymized data not published within this article will be made available by request from any qualified investigator.

## Abbreviations

BVMT-R: Brief Visuospatial Memory Test–Revised
CI: Cognitively impaired
CIS: Checklist Individual Strength
CP: Cognitively preserved
EDSS: Expanded Disability Status Scale
HC: Healthy controls
HPT: 9-Hole Peg Test
LV: Lesion volume
MFIS: Modified Fatigue Impact Scale
MS: Multiple sclerosis
nCortexV: Normalized cortical volume
nTBV: Normalized total brain volume
nThalV: Normalized thalamus volume
pwMS: People with multiple sclerosis
SDMT: Symbol digit modalities test
sSDMT: Smartphone SDMT
T25FW: Timed 25-Foot Walk
VLGT: Verbal Learning and Memory Test (Dutch translation of the CVLT-II)
