# Supplementary material for: The practice effect of smartphone-derived cognitive processing speed assessments as a proxy of cognitive functioning in multiple sclerosis
Source: J Neurol. 2026 May 7;273(6):305. doi: 10.1007/s00415-026-13835-9 (PMC13152975; doi:10.1007/s00415-026-13835-9)
Supplement: Supplementary file 1 — Supplementary file1 (DOCX 4262 KB) [file 415_2026_13835_MOESM1_ESM.docx]

**Supplementary Methods S1:** MRI Acquisition and Processing

*MRI Acquisition*

HC and pwMS underwent MRI acquisitions at baseline using a 3T GE Discovery MR750 (GE Healthcare, Milwaukee, USA) with an 8-channel head coil. The protocol included a 3D-T1-weighted (T1w) fast spoiled gradient-echo sequence (repetition time 8.2ms, echo time 3.2ms, inversion time 450ms, flip angle 12°, voxel size: 0.9×0.9×1.0mm^3^) and 3D-Fluid-Attenuated Inversion Recovery (FLAIR: repetition time 8000ms, echo time 129ms, inversion time 2340ms, voxel size 1.0×1.0×1.2mm^3^). For pwMS with relapsing-remitting MS a post-gadolinium contrast-enhanced axial 2D-T1w image (repetition time 600ms, echo time 8ms, flip angle 125°, FOV 250mm, voxel size: 0.5x0.5x5.0mm^3^) was also acquired at baseline and 3 months after inclusion.

*MRI Processing*

MRI analyses were previously described in detail.^1^ In short, on the baseline T1w and FLAIR, white matter hyperintensities were automatically segmented using scanner-optimised nicMSLesions (v0.2).^2^ Lesion masks were manually corrected if necessary. White matter hyperintensities were lesion-filled on the T1w using NiftySeg based on the lesion masks.^3^ Thereafter, Sequence Adaptive Multimodal SEGmentation (SAMSEG, FreeSurfer v7.3.2) was applied on the lesion-filled T1w scan to obtain the total brain, cortex, and thalamic volumes.^4^ Ratio-based normalisation was performed using the segmentation-based total intracranial volume (sbTIV) resulting in normalised total brain volume (nTBV), normalised cortex volume (nCortexV), and normalised thalamic volume (nThalV). The total lesion volume (LV) was not normalised.

1. Molenaar PCG, Noteboom S, van Nederpelt DR, et al. Digital outcome measures are associated with brain atrophy in patients with multiple sclerosis. *J Neurol* 2024 doi: 10.1007/s00415-024-12516-9 [published Online First: 20240715]

2. van Nederpelt DR, Pontillo G, Barrantes-Cepas M, et al. Scanner-specific optimisation of automated lesion segmentation in MS. *Neuroimage Clin* 2024;44:103680. doi: 10.1016/j.nicl.2024.103680 [published Online First: 20241002]

3. Prados F, Cardoso MJ, Kanber B, et al. A multi-time-point modality-agnostic patch-based method for lesion filling in multiple sclerosis. *Neuroimage* 2016;139:376–84. doi: 10.1016/j.neuroimage.2016.06.053 [published Online First: 20160701]

4. Puonti O, Iglesias JE, Van Leemput K. Fast and sequence-adaptive whole-brain segmentation using parametric Bayesian modeling. *Neuroimage* 2016;143:235–49. doi: 10.1016/j.neuroimage.2016.09.011 [published Online First: 20160907]

**Supplement Figure S1**: Logarithmic function to determine practice effects


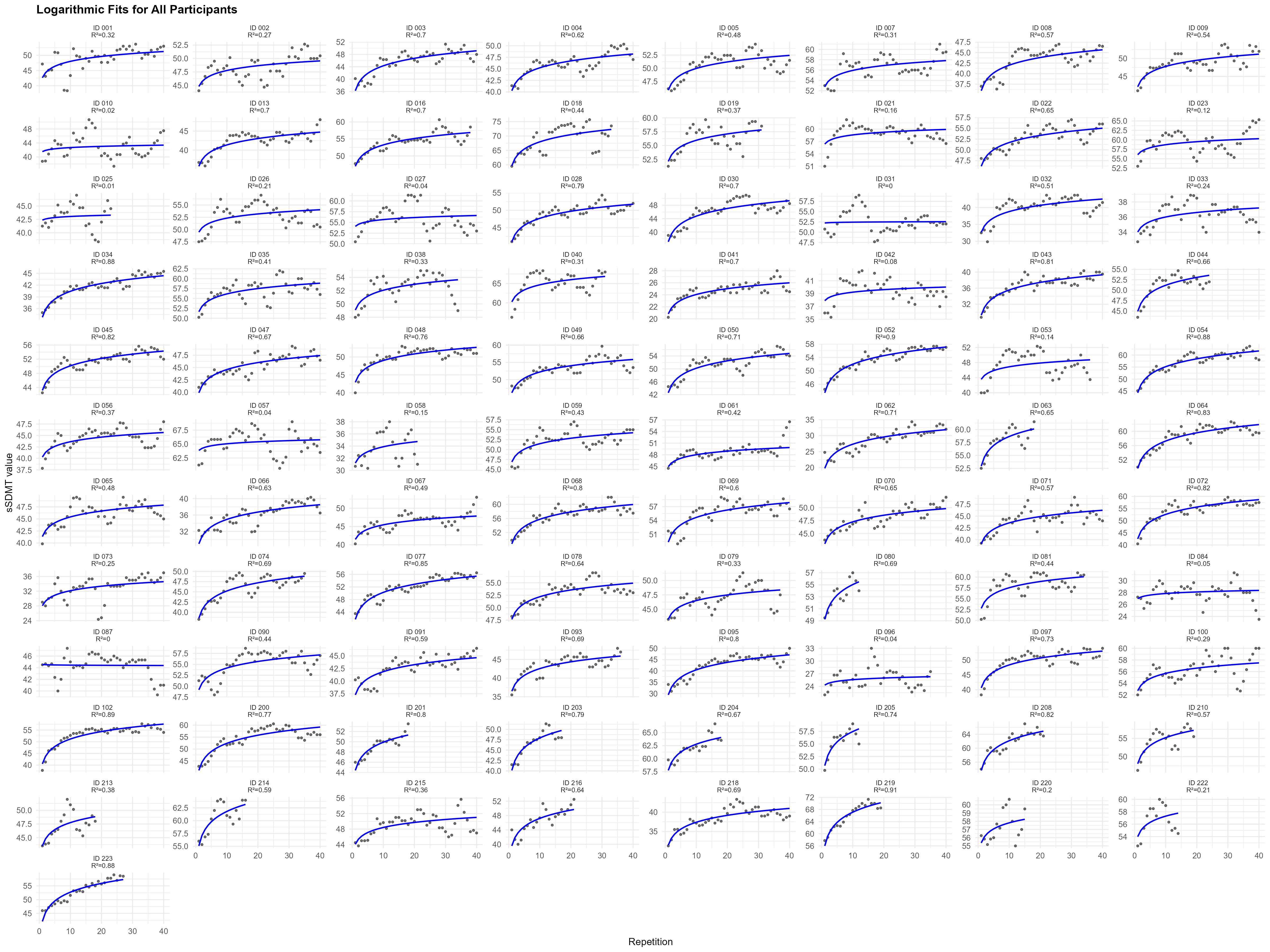


Supplement Figure S1: A logarithmic function ($y=a+b*log(repetition)$) fit to the sSDMT repetition data (R^2^=0.520) for all participants that reached a plateau using the breakpoint linear regression. The resulting variables are the baseline sSDMT (variable a) and a variable indicating the degree of practicing (variable b).

**Supplement Figure S2**: Inverse exponential function to determine practice effects


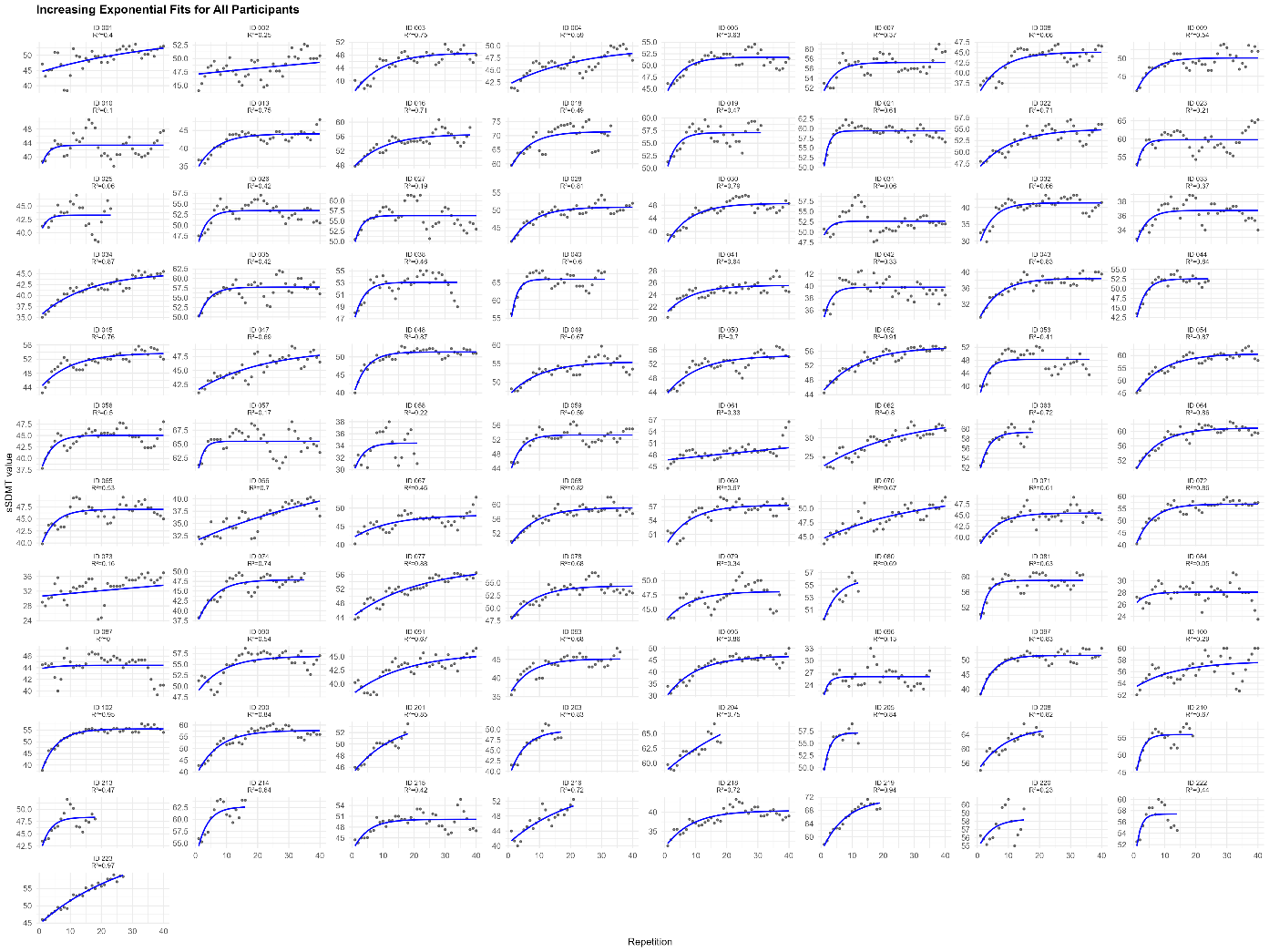


Supplement Figure S2: An inverse exponential ($y=a+b*\left( 1-\left( -c*repetition \right)^{2} \right)$) fit to the sSDMT repetition data (R2=0.587) for all participants that reached a plateau using the breakpoint linear regression. The resulting variables are the baseline sSDMT (variable a), the total increase in sSDMT score (variable b), and a variable indicating the degree and speed of practicing (variable c).

**Supplement Figure S3**: Spearman correlation matrix of practice effect outcomes


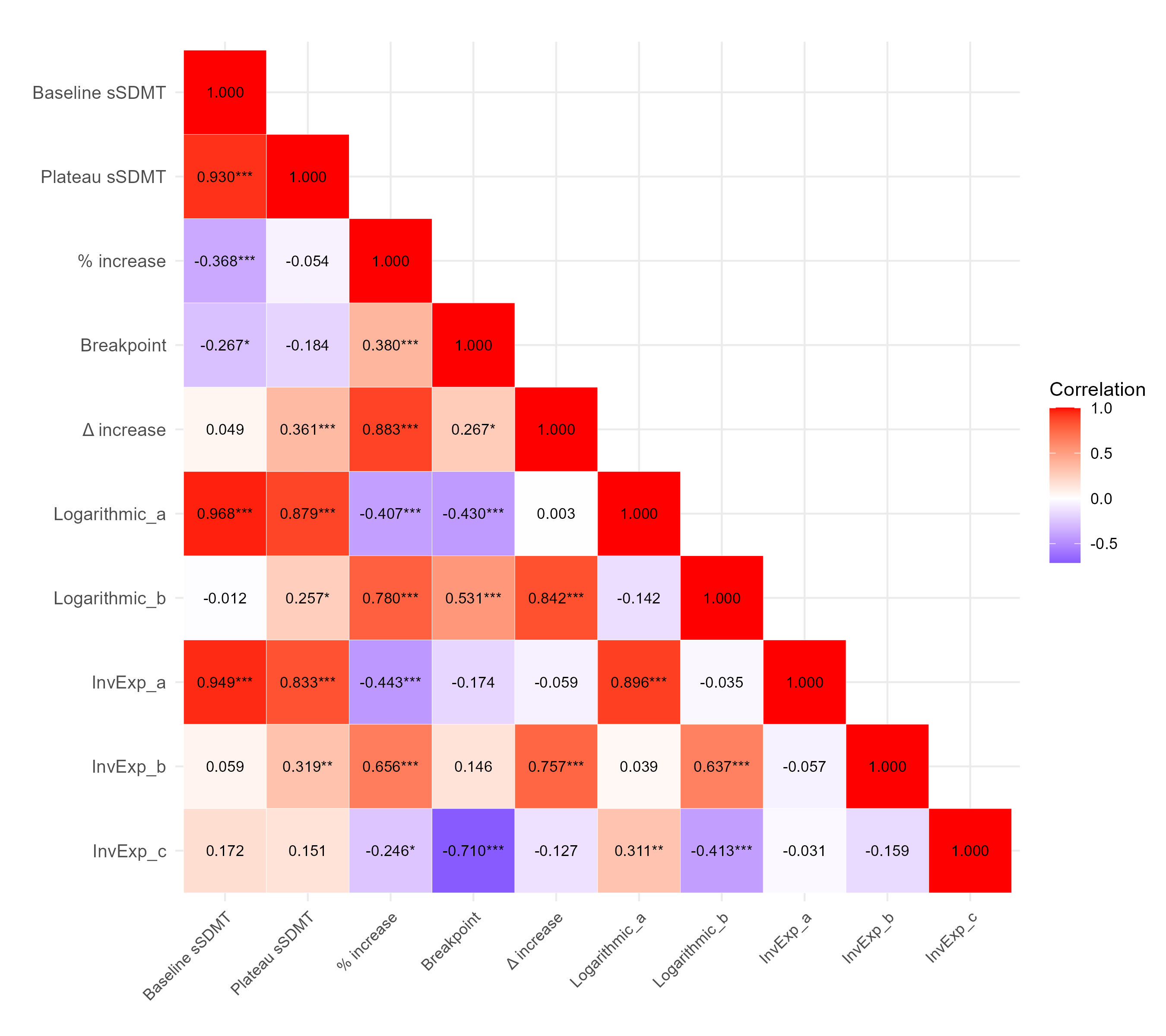


Supplement Figure S3: Spearman correlation matrix of practice effect outcomes of the breakpoint linear regression (baseline sSDMT, plateau sSDMT, Δ increase, % increase, Breakpoint), logarithmic (Logarithmic_a and Logarithmic_b) and inverse exponential functions (InvExp_a, InvExp_b, InvExp_c). Stars indicate statistical significance: *** p <0.001, ** p<0.01, * p<0.05.

**Supplement Table S1:** Practice effect outcomes of different non-linear models in relation to the EDSS

| **Practice effect outcome** | **Std. β** | **95% CI** | **p value** |
| --- | --- | --- | --- |
| Baseline sSDMT | -0.078 | (-0.506 – -0.417) | 0.001 |
| Plateau sSDMT | -0.056 | (-0.399 – -0.319) | 0.007 |
| % increase | 0.000 | (0.183 – 0.183) | 0.126 |
| Breakpoint | 0.029 | (0.079 – 0.184) | 0.272 |
| Δ increase | 0.027 | (-0.058 – 0.169) | 0.631 |
| Logarithmic_a | -0.072 | (-0.502 – -0.419) | 0.001 |
| Logarithmic_b | 0.100 | (-0.209 – 0.368) | 0.491 |
| InvExp_a | -0.080 | (-0.508 – -0.423) | 0.000 |
| InvExp_b | -0.005 | (-0.084 – 0.046) | 0.868 |
| InvExp_c | 0.315 | (-2.046 – 2.121) | 0.764 |
|  |  |  |  |

Supplementary Table S1: Outcomes of univariable linear regressions between individual practice effect-curve parameters from different non-linear models (breakpoint linear regression, logarithmic, inverse exponential) and EDSS, corrected for age and sex. Values represent standardised regression coefficients (Std. β), with corresponding 95% confidence intervals and p-values.

**Supplementary Analyses S1:** Assessment of bias caused by missing data among pwMS

Because we used a per case exclusion of cases with missing data for the analyses, analyses might be biased by missingness of the data. Therefore, to assess this bias we examined to what extent cases with missing data systematically differed from cases with complete data. First, we mapped missing data per pwMS in *Supplementary Analyses S1* ***-*** *Figure 1* and in *Supplementary Analyses S1* ***–*** *Table 1*. Finally, two groups of variables with >5% of missing data (MRI variables at baseline, 5Y clinical outcomes) were assessed for systematic differences between missing and non-missing data. This was done using T-tests, Mann-Whitney U, and Chi-square tests. The results, see *Supplementary Analyses S1* ***–*** *Table 2,* indicated that for these two groups of variables, no systematic differences were found between complete cases and cases with missing data.


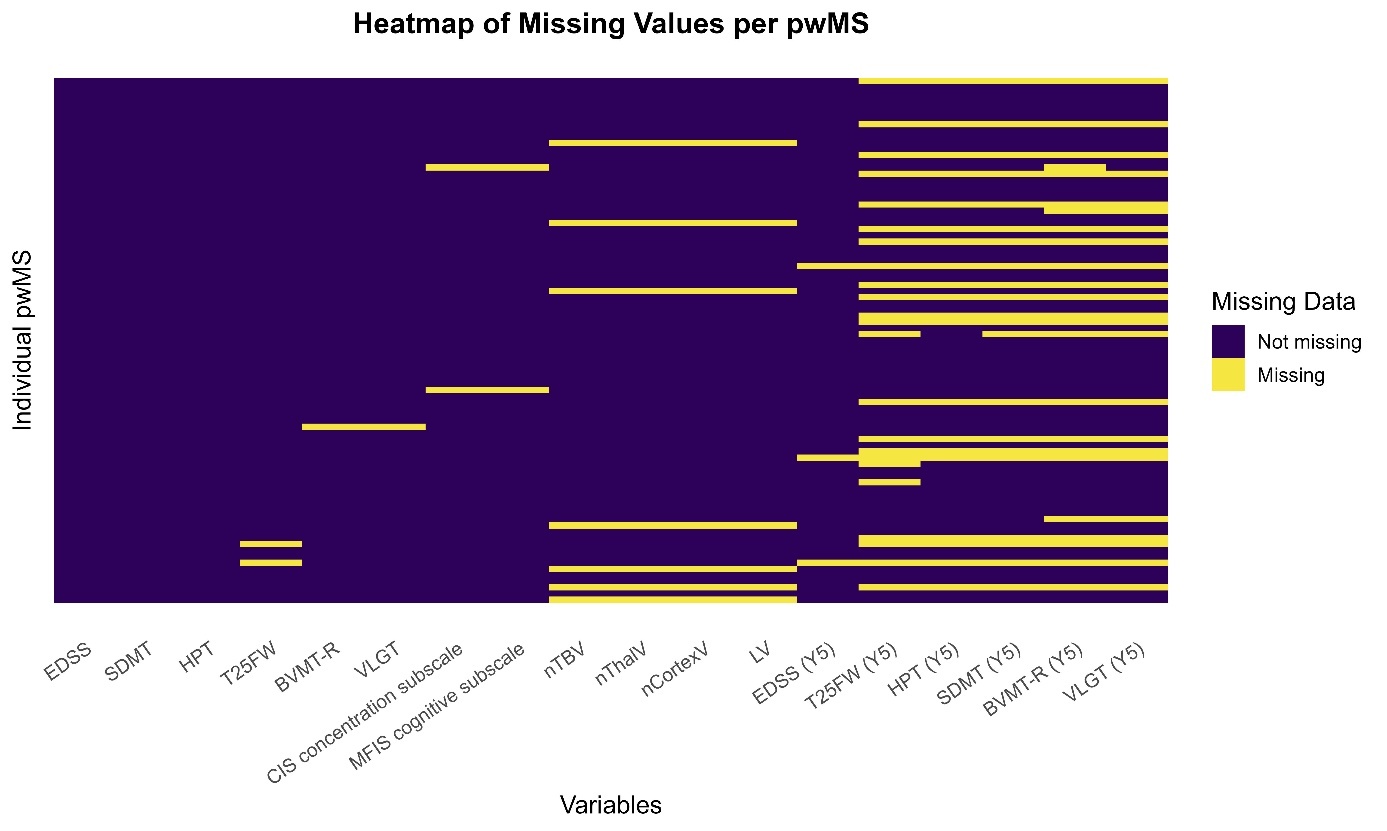


*Supplementary Analyses S1* ***-*** *Figure 1:* Heatmap of missing Values per pwMS.

| **Variable** | **Missing %** |
| --- | --- |
| ***Clinical variables*** |  |
| EDSS | 0 |
| SDMT | 0 |
| HPT | 0 |
| T25FW | 2.4 |
| BVMT-R | 1.2 |
| VLGT | 1.2 |
| ***Patient-reported outcomes*** |  |
| CIS concentration subscale | 2.4 |
| MFIS cognitive subscale | 2.4 |
| ***MRI variables*** |  |
| nTBV | 8.2 |
| nThalV | 8.2 |
| nCortexV | 8.2 |
| LV | 8.2 |
| ***Clinical variables at 5Y follow-up*** |  |
| EDSS (Y5) | 3.5 |
| T25FW (Y5) | 27.1 |
| HPT (Y5) | 23.5 |
| SDMT (Y5) | 24.7 |
| BVMT-R (Y5) | 28.2 |
| VLGT (Y5) | 27.1 |
|  |  |

*Supplementary Analyses S1* ***–*** *Table 1:* Percentage of the pwMS with missing data per variable.

| **MRI variables (8.2% missing)** | |  |  |
| --- | --- | --- | --- |
| **Variable** | **Missing cases** | **Complete cases** | **p value** |
| Age | 47.9 (9.9) | 45.4 (10.3) | 0.566 |
| Sex | male: 23 / female: 55 | male: 2 / female: 5 | 1.000 |
| Disease duration | 6.3 [2.9, 12.9] | 7.3 [5.2, 13.1] | 0.448 |
| EDSS (baseline) | 3.5 [2.5, 4.0] | 3.5 [3.0, 4.2] | 0.815 |
| SDMT | 54.4 (9.9) | 50.7 (11.5) | 0.435 |
|  |  |  |  |
| **5Y clinical outcomes (30.6% missing)** | |  |  |
| **Variable** | **Missing cases** | **Complete cases** | **p value** |
| Age | 47.8 (10.0) | 47.6 (9.9) | 0.915 |
| Sex | male: 6 / female: 20 | male: 19 / female: 40 | 0.553 |
| Disease duration | 5.1 [3.3, 10.9] | 7.3 [3.0, 14.1] | 0.448 |
| EDSS (baseline) | 3.8 [2.6, 4.5] | 3.5 [2.8, 4.0] | 0.378 |
| SDMT | 52.3 (9.5) | 54.9 (10.2) | 0.250 |

*Supplementary Analyses S1* ***–*** *Table 2:* Analyses of structural differences between cases with missing and non-missing data. Analyses were performed using T-tests, Mann-Whitney U, and Chi-square tests.

**Supplement Analysis S2**: Analysis of CI in relation to the HPT

To evaluate the extent to which the arm function influences the sSDMT test result, we evaluated the relationship between the HPT and the CI/CP classification. We found that the relationship between the baseline sSDMT value and the cognitive status at baseline (CP: β=13.71, p<0.001) was confounded by the HPT (CP: β=11.02, p<0.001) and as 9.2% of the variance was explained by the HPT. However, after trimming the 10 highest HPT values (CP: 5, CI: 5) the HPT was no longer a confounder. Therefore, we proceeded with our analyses without correcting for the HPT since the effect was driven by the 10 pwMS with the highest HPT, these pwMS were evenly distributed, and HPT is a measure of disability.

**Supplement Figure S4**: Curves of participants without a quantifiable practice effect


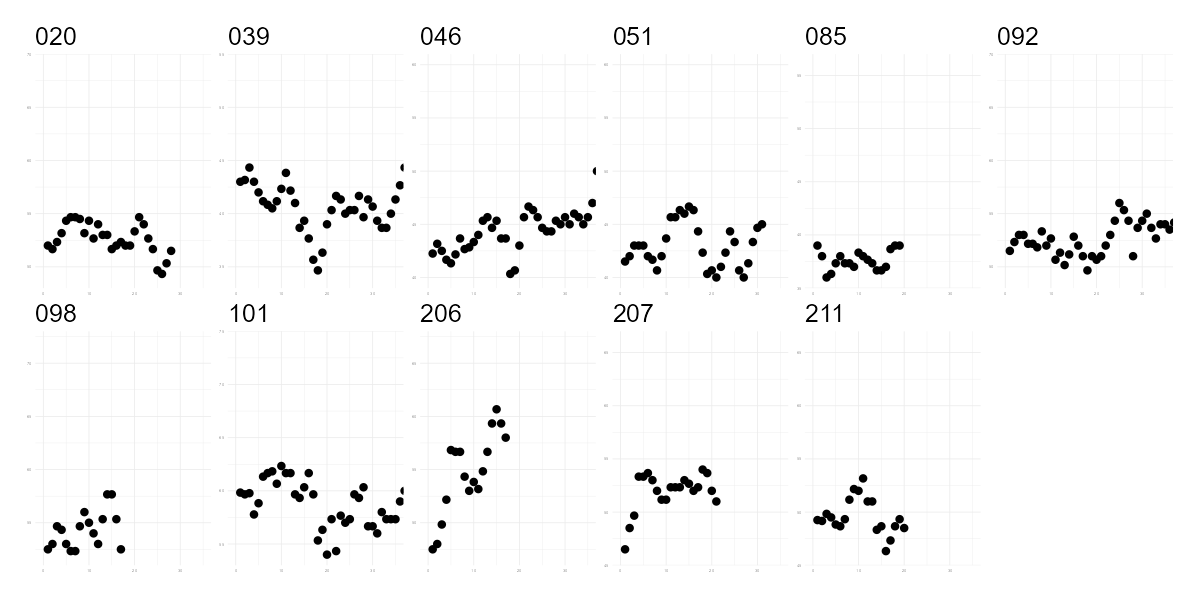


Supplement Figure S6: Curves of people wherein no plateau could be identified and that had at least 15 repetitions on the sSDMT. Participants 020-101 are people with MS and were all considered cognitively preserved at baseline. Participants 206-211 are healthy controls.

**Supplement Figure S5**: Comparison between HC, CP, and CI using the plateau sSDMT for CI definition


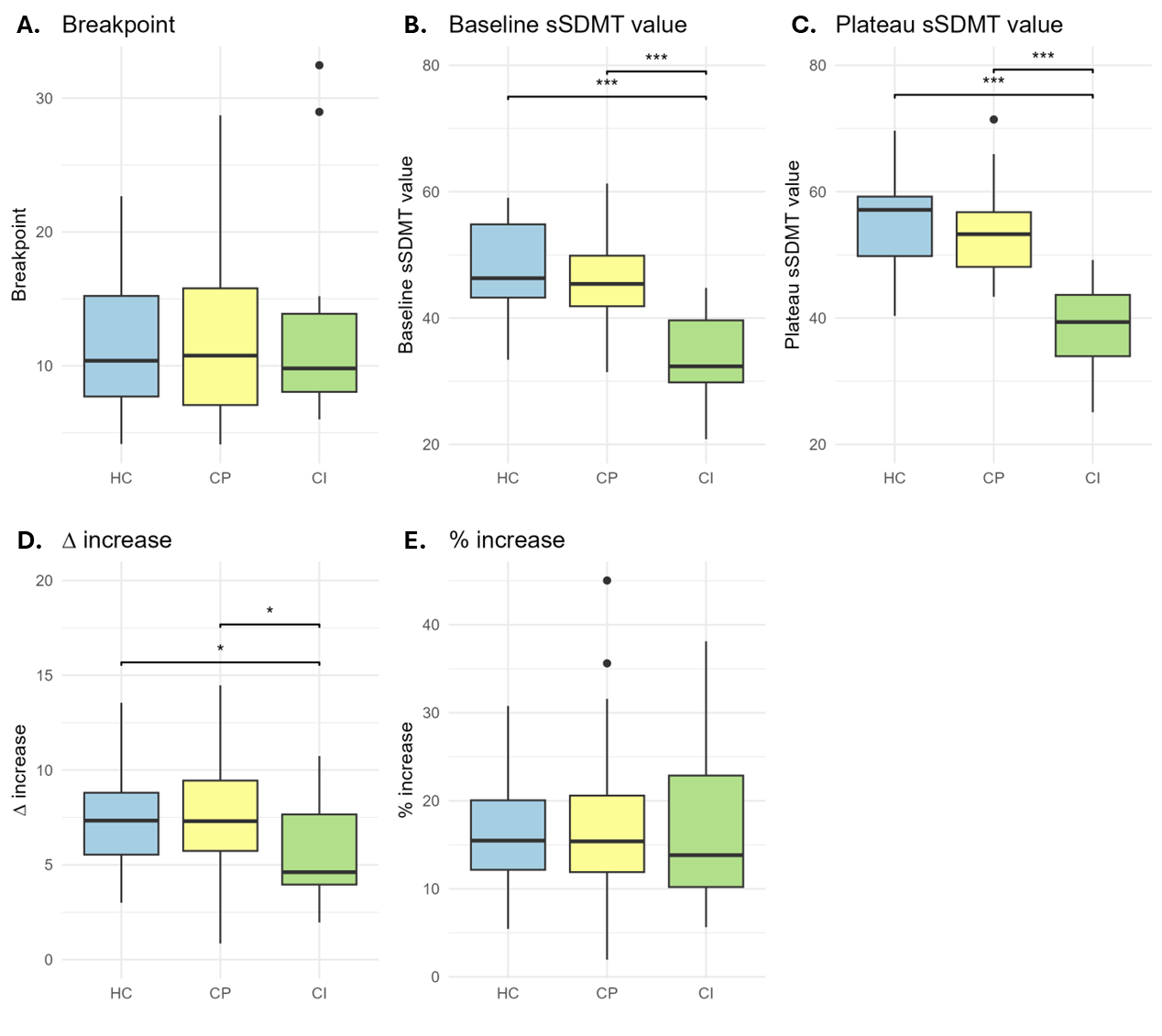


Supplement Figure S4: Depicted are boxplots (median and IQR) extracted variables of the individual level practice effect curves stratified into healthy controls (HC), cognitively preserved (CP), and cognitively impaired (CI) based on the plateau sSDMT outcome. Depicted are (**A**) the amount of repetitions until the breakpoint is reached (breakpoint repetition), (**B**) baseline sSDMT level, (**C**) plateau sSDMT level, (**D**) delta increase (Δ increase), and (**E**) the % increase (Δ increase /baseline sSDMT level). The practice outcomes are compared using an ANCOVA with Tukey HSD-adjusted post-hoc comparisons, adjusted for age, sex, education, and days until reaching a plateau. Only statistically significant results are depicted wherein *adj.p < 0.05, **adj.p < 0.01, and ***adj.p < 0.001.

**Supplemental Figure S6:** CI/CP classification using the baseline vs the plateau sSDMT excluding non-plateau HC and pwMS


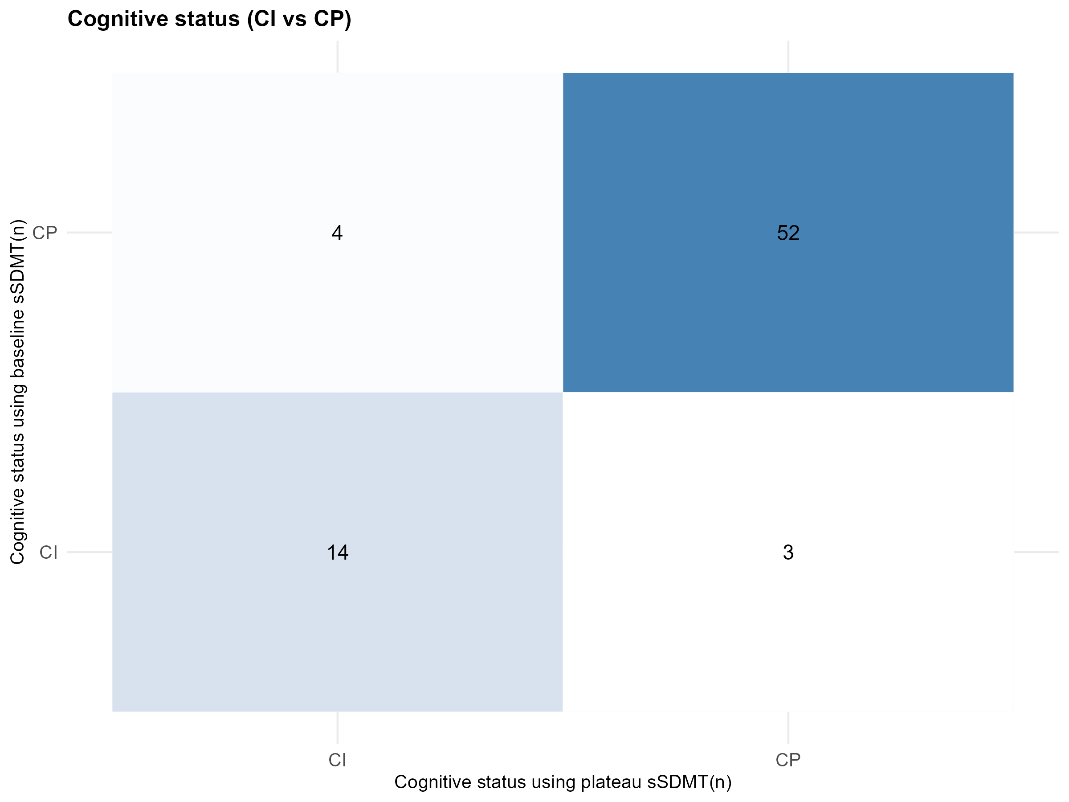


Supplemental Figure S5: CI/CP classification using either the baseline sSDMT or the plateau sSDMT excluding non-plateau HC and pwMS. The mean z-score for the baseline sSDMT was z=-0.679±1.31 and for the plateau sSDMT z=-0.664±1.53.
